# Exploring the evolution of multicellularity in *Saccharomyces cerevisiae* under bacteria environment: An experimental phylogenetics approach

**DOI:** 10.1002/ece3.3979

**Published:** 2018-04-15

**Authors:** Julian F. Quintero‐Galvis, Rocío Paleo‐López, Jaiber J. Solano‐Iguaran, María Josefina Poupin, Thomas Ledger, Juan Diego Gaitan‐Espitia, Andrzej Antoł, Michael Travisano, Roberto F. Nespolo

**Affiliations:** ^1^ Instituto de Ciencias Ambientales y Evolutivas Universidad Austral de Chile Valdivia Chile; ^2^ Center of Applied Ecology and Sustainability (CAPES‐UC) Facultad de Ciencias Biológicas Universidad Católica de Chile Santiago Chile; ^3^ Laboratorio de Bioingeniería Facultad de Ingeniería y Ciencias Universidad Adolfo Ibáñez Santiago Chile; ^4^ The Swire Institute of Marine Science and School of Biological Sciences The University of Hong Kong Hong Kong China; ^5^ CSIRO Oceans & Atmosphere Hobart TAS Australia; ^6^ Institute of Environmental Sciences Jagiellonian University Kraków Poland; ^7^ Department of Ecology, Evolution and Behavior University of Minnesota Minneapolis MN USA; ^8^ Millennium Institute for Integrative Systems and Synthetic Biology (MIISSB) Santiago Chile

**Keywords:** clonal multicellularity, experimental evolution, multicellularity, social multicellularity, yeasts

## Abstract

There have been over 25 independent unicellular to multicellular evolutionary transitions, which have been transformational in the complexity of life. All of these transitions likely occurred in communities numerically dominated by unicellular organisms, mostly bacteria. Hence, it is reasonable to expect that bacteria were involved in generating the ecological conditions that promoted the stability and proliferation of the first multicellular forms as protective units. In this study, we addressed this problem by analyzing the occurrence of multicellularity in an experimental phylogeny of yeasts (*Sacharomyces cerevisiae*) a model organism that is unicellular but can generate multicellular clusters under some conditions. We exposed a single ancestral population to periodic divergences, coevolving with a cocktail of environmental bacteria that were inoculated to the environment of the ancestor, and compared to a control (no bacteria). We quantified culturable microorganisms to the level of genera, finding up to 20 taxa (all bacteria) that competed with the yeasts during diversification. After 600 generations of coevolution, the yeasts produced two types of multicellular clusters: clonal and aggregative. Whereas clonal clusters were present in both treatments, aggregative clusters were only present under the bacteria treatment and showed significant phylogenetic signal. However, clonal clusters showed different properties if bacteria were present as follows: They were more abundant and significantly smaller than in the control. These results indicate that bacteria are important modulators of the occurrence of multicellularity, providing support to the idea that they generated the ecological conditions‐promoting multicellularity.

## 1 INTRODUCTION

A salient fact of any interpretation of the modern tree of life is that eukaryotic life appeared at least a billion years after the first prokaryotic organism (Hug et al., 2016). Then, the multiple transitions of unicellular to multicellular lifestyle that have been described should have occurred in communities numerically dominated by bacteria (Alegado & King, 2014). Bacteria represent a selective pressure as they modify the ecological conditions other organisms perceive as follows: They compete for nutrients, they generate toxic compounds, and they acidify the medium (Alegado & King, 2014; Theobald, 2010; Viljoen, 2001). In fact, comparative evidence suggests that the last common ancestor of animals (Metazoans) had structures for consuming bacteria (collar cells) and also defensive proteins and domains against them (Alegado & King, 2014). In choanoflagellates, the closest living relatives of animals, cell‐to‐bacteria interactions are known to induce other defensive responses, for example, the formation of small colonies, highlighting the possibility that these interactions were fundamental during the evolution of animal multicellularity (Alegado et al., 2012; Fairclough, Dayel, & King, 2010). While these examples suggest that unicellular bacteria can play important roles affecting multicellular traits, experimental evidence showing how multicellularity arise as a response to bacteria is lacking.

Identifying the long‐term benefits of multicellularity is relatively simple (e.g., division of labor, functional specialization, biological complexity; see reviews in Celiker & Gore, 2013; Grosberg & Strathmann, 2007), and explains why this feature appeared so many times in the tree of life, representing a major transition in evolution (Duran‐Nebreda & Sole, 2015; Grosberg & Strathmann, 2007). However, reproducing the ecological conditions of promoting the stability and proliferation of the first multicellular clusters have proved to be challenging. For instance, the experimental evolution of multicellularity in yeasts suggests that fast sinking (cell size, ultimately) is the primary criterion to produce clonal clusters (“snowflakes,” see Ratcliff, Denison, Borrello, & Travisano, 2012). In these experiments, snowflakes appear spontaneously in liquid laboratory growing conditions and respond rapidly to settling selection, increasing settling speed, mean size and cluster complexity after a few hundreds of generations (Ratcliff, Fankhauser, Rogers, Greig, & Travisano, 2015; Ratcliff et al., 2012). These evolved snowflakes display a number of important emergent properties: They grow and reproduce after a critical size, they settle rapidly enough to survive, or fail to do so and perish (Ratcliff et al., 2015). These unique experiments addressed central evolutionary questions (e.g., origin of life histories, labor repartition, and biological complexity) that would be very difficult to study using other approaches (e.g., retrospective or comparative analyses, see Herron, 2016). Moreover, these studies also demonstrate how easy to evolve is multicellularity in an organism that is normally unicellular.

Many microorganisms develop transitory multicellular aggregations as protective devices, such as biofilms, filaments, or fruiting bodies that can persist for several generations. These “social” or “aggregative” structures originate as a response to a broad array of stimuli (e.g., chemical stress, starvation, and defense) and are functionally different to the previously described clonal multicellularity because aggregative clusters are genetically diverse as they result from the association among different cell lineages (Claessen, Rozen, Kuipers, Sogaard‐Andersen, & van Wezel, 2014; Grosberg & Strathmann, 1998, 2007; Kuthan et al., 2003; Ratcliff et al., 2015; Veelders et al., 2010). The development of aggregates in wild strains of *Sacharomyces cerevisiae* is also known as flocculation, a metabolic strategy for survival under unfavorable conditions (Kuthan et al., 2003). Smukalla et al. (2008) described in the S288C strain that the cooperative behavior of *S. cerevisiae* is controlled by a multigene family at subtelomeric localization (i.g., *FLO1*,* FLO5*, and *FLO8;* see Smukalla et al., 2008; Teunissen & Steensma, 1995) that promotes aggregation of cells carrying the same mutation, and showed that this capacity is highly variable among strains, suggesting this is a rapidly evolving trait (Pentz, Travisano, & Ratcliff, 2014; Smukalla et al., 2008).

The evidences discussed above had led some authors to propose that bacteria can generate environmental conditions promoting a multicellular lifestyle (see McFall‐Ngai et al., 2013; Woznica et al., 2016). This idea comes from two main lines of observations. The first is comparative: Several animal larvae (e.g., sponges, cnidarians, bryozoans, and ascidians, reviewed in Alegado & King, 2014; McFall‐Ngai et al., 2013) settle in response to bacterial chemical cues, suggesting that bacteria are involved in their uni‐ to multicellular life history shift, as an inherited feature of unicellular ancestors of animals. A second line of evidence is experimental: Some organisms that develop facultative multicellularity (e.g., choanoflagellates) generate clonal aggregations in the presence of bacteria (Alegado et al., 2012; Woznica et al., 2016). Bacteria also influence the life history of other microorganisms such as *Dictyostelium discoideum* (Adu‐Oppong, Queller, & Strassmann, 2014) or *Myxococcus xanthus* which feed on bacteria and produce fruiting bodies (i.e., social multicellularity) when they are scarce (see also Celiker & Gore, 2013).

In this paper, we explored how bacteria interact with populations of *S. cerevisiae*, using a system in which multicellular traits reliably and repeatedly evolve. We anticipated that if bacteria represent a strong selective pressure on the transition to multicellularity, then those effects would be reflected in an experimental diversification of lineages. To mimic the cladogenetic nature of macroevolution, our experiment consisted in periodically imposing population splits to a focal populations of *S. cerevisiae*, for a total of 600 generations, thus reproducing a “micro‐phylogeny” of lineages (e.g., Bull, Cunningham, Molineux, Badgett, & Hillis, 1993; Hillis, Bull, White, Badgett, & Molineux, 1992; Oakley & Cunningham, 2000; Oakley, Gu, Abouheif, Patel, & Li, 2005). Here (and elsewhere), we operationally use the term “experimental phylogeny” for this setup, but we hasten to indicate this is only an operational definition. Interestingly, our results show that aggregative multicellular forms (=social multicellularity) were produced in the presence of bacteria, in some lineages, producing a significant phylogenetic signal. Also, clonal multicellularity was frequent in both treatments, but was not statistically associated with the bacteria treatment. Thus, it seems that yeasts react defensively to bacteria, producing large multicellular clusters.

## 2 METHODS

### 2.1 Experimental phylogeny

**Figure 1:**
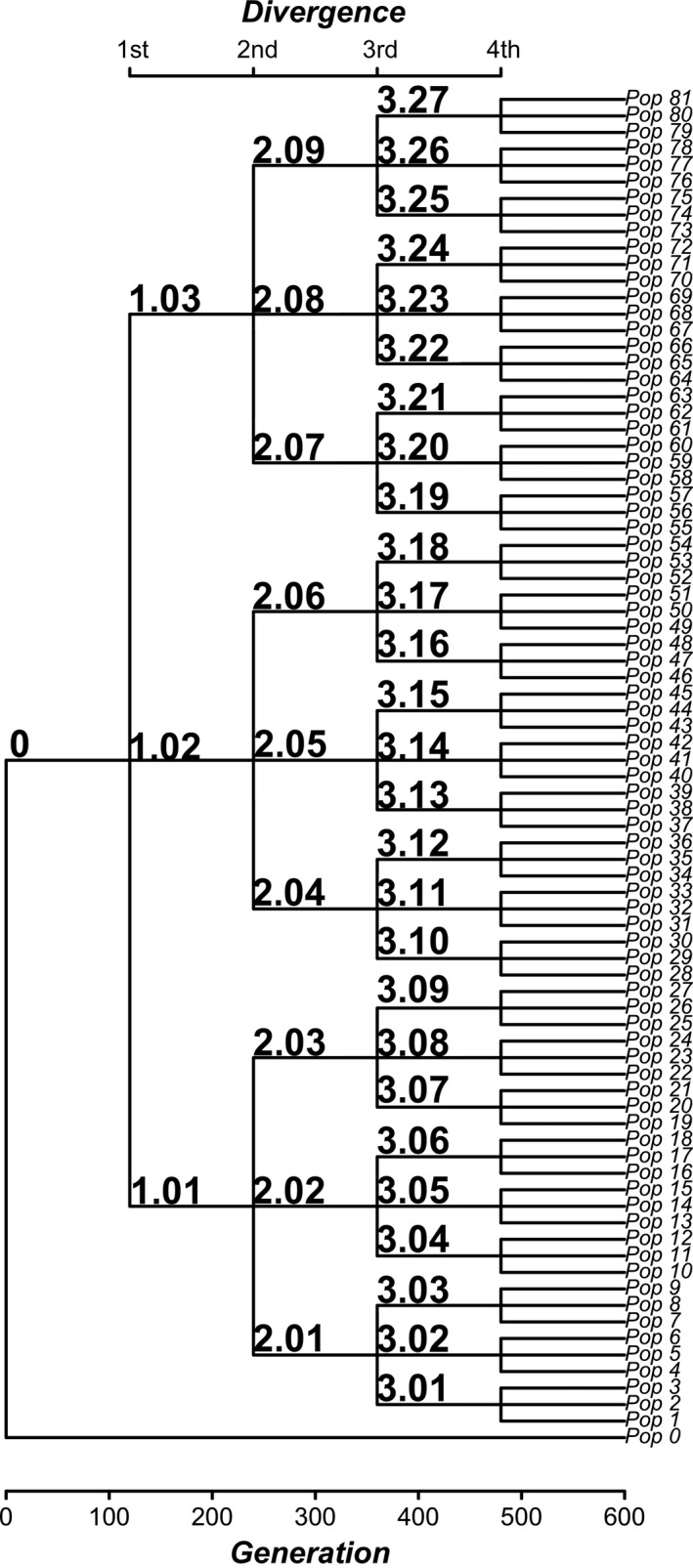
An experimental phylogeny of lineages of approximately 600 generations. The ancestor (population 0) was generated from a spore print of the Y55 strain, and let to evolve with daily transfers during approximately 120 generations (3 weeks) previously to the first divergence. This first divergence produced populations 1.01, 1.02, and 1.03 which were evolved other 120 generations, and then split in three again (producing populations 2.01–2.09), evolved and split again during 120 generation, and producing 3.01–3.27; and evolved again and split as before, producing the 81 final “tips” (“Pop 1” to “Pop 81”). A single population was maintained without divergences, as an “outgroup” (denoted as “Pop 0”). The bacteria treatment was a similar protocol excepting that the ancestor was left to be colonized with environmental bacteria (see Section 2 for details)

We used an auxotrophic, diploid, and homothallic strain Y55 (*Mat a*/α*,* URA3*::KanMX*) (Cubillos, Louis, & Liti, 2009; Herskowitz, 1988), which is ideal for evolutionary studies of diversification because of its relatively undomesticated nature, and it also can be evolved to multicellularity (see below). We initiated the experiments with one ancestral population that was previously sporulated for increasing genetic variation, using the standard chloroform method, and printed in a sterilized piece of paper. Then, we inoculated this spore print into a 10 × 100 mm glass culture tube containing 3.5 ml of nonsterilized yeast peptone dextrose media (YPD: 1% yeast extract, 2% peptone, and 2% dextrose). This procedure allowed the colonization of the spore print with environmental bacteria. The culture tube was maintained at 30°C in an orbital shaker at 150 rpm. Every day, 50 μl of the experimental culture (yeast and bacteria) was transferred to a new tube with fresh sterilized YPD media. These daily transfers were maintained until the end of the experiments (~600 generations, ~3 months). We split the culture every 21 days (~120 generations) leading to a total of four divergence events (Figure 1). One tube was maintained as an “outgroup,” without divergence (i.e., Pop 0, Figure 1). With this protocol, we generated an experimental diversification (of lineages; our “experimental phylogeny”) with 81 tips, 40 internal nodes, and constant branch lengths, following phylogenetic notation (Paradis, 2012). Samples were observed at the microscope weekly, finding some bacteria approximately at the second divergence (this experiment proceeded until the fourth divergence, see Figure 1). A second experiment (i.e., the “control”) was performed, where the appearance of bacteria was strictly controlled. To attain this, the experiment was repeated exactly as described before, but applying a small concentration of antibiotics for which the Y55 is resistant (10 μl/ml Ampicillin; 4 μl/ml; Rifampicin and Tetracycline). The antibiotics were applied at the beginning of each divergence (i.e., four times), and were maintained only during one transfer (i.e., 24 hr; ~six generations). To confirm that only the focal organisms was measured (*S. cerevisiae*), the following procedures were applied regularly: (1) all possible forms of life were detected by plating and by PCR methods (see below), (2) we performed exhaustive observations using the optic microscope and the Neubauer chamber (see below), and (3) we plated the cultures and the isolated yeast colonies were grown in YPD and identified by microscopy.

### 2.2 Bacterial isolation and quantification

In order to quantify bacterial abundance across divergences, a sample of 250 μl of the media was taken randomly immediately after each diversification (according to Figure 1). Given that we were focused on the yeasts, a culture‐dependent technique was used to generate a general overview of the bacterial diversity across the experiment. Culture‐based techniques are a reliable approach to characterize bacterial populations in a sample, but have selectivity for abundant and culturable taxa (Amann, Ludwig, & Schleifer, 1995; Pace, 1997). Nevertheless, some studies have reported that many of the proportionally dominant taxa identified by culture‐independent methods (i.e., pyrosequencing) could also be found by isolation (Bodenhausen, Horton, & Bergelson, 2013; Jackson, Randolph, Osborn, & Tyler, 2013).

The sample was diluted in one volume of YPD liquid media, and recovered after 2 hr at room temperature without shaking. Then, samples were serially diluted in sterile 10 mmol/L MgSO_4_, and plated on YPD, R2A, diluted (1/5) LB agar, TSA and TS‐blood agar media in three replicates. The plates were incubated at 28 ± 2°C until the appearance of microbial colonies. Individual colonies were picked and streaked on the different media for further characterization. Colonies were classified and quantified by morphology, size, color, shape, growth pattern and Gram staining, and cell sizes and shapes were observed by light microscopy. Based on these phenotypic observations, twenty‐two bacterial types were clearly distinguishable and representative cultures of each type were preserved for genetic characterization. From each culture, we estimated bacteria abundance based on the number of colony‐forming units, correcting by the dilution factor. This is a rough approximation to a density (i.e., cells/μl), as each colony was initiated by a single cell. Later (see below), these quantification were assigned to a given taxa (Table S1).

Total genomic DNA of the bacterial strains was extracted using the Wizard^®^ Genomics DNA purification kit (Promega). Purified DNA was subject to 16S rRNA gene amplification using the forward primer: 27F (5′ AGA GTT TGA TCC TGG CTC AG 3′) and the reverse primer: 1492R (5′ CGG CTA CCT TGT TAC GAC TT 3′) as described in Amann et al. (1995). Briefly, the PCR mixture (25 μl) contained 20 ng of template DNA, 200 nmol/L of each primer, 1.5 mmol/L of MgCl2, and 0.5 mol/L Betaine. Amplification was performed under the following conditions: 95°C for 10 min, followed by 25 cycles of 94°C, 45 s; 56°C, 45 s; and 72°C, 2 min, with a final extension of 7 min at 72°C (Amann et al., 1995). The quality of the PCR products was confirmed by gel electrophoresis, and the amplified products were purified using a QIAquick PCR Purification kit (QIAGEN). Sequences were obtained from Macrogen Sequencing service (Macrogen, Korea), using the primers mentioned above. Taxonomic assignments were obtained from nucleotide alignments (BLASTn, NCBI) of the complete 1,465 bp sequence obtained for each bacterial type, using a 97% identity threshold against the database of the 16s sequence for each bacteria.

### 2.3 Identification of clonal and aggregative clusters

In order to characterize the multicellular clusters in the daughter populations (i.e., the tips of the tree in Figure 1), 50 μl of each population was transferred to fresh medium and let to grow during 24 hr in the same conditions as during the diversification. After 24 hr of growth, 10 μl of cell culture was diluted in 100 μl of YPD medium (1:10) and 10 μl of diluted sample was loaded onto a Neubauer chamber (Hirschmann Laborgeraete GmbH & Co, Germany). Two samples were put into two chambers on one slide, and treated as technical replicates. Photographs were taken using a Motic BA 310 microscope with a Canon Reflex 5 camera (10× and 40× magnification). At least 10 photographs were taken from each population: Two under the 10× objective and eight under the 40× objective. The first photographs were used for covering the central part of the gridding chamber, and whenever multicellular clusters were identified outside the central grid, additional 10× photographs were made covering the whole slide. 40× photographs were taken in the four smaller grids at the central section of the slide and also outside of the grid if more clusters were detected. In the bacteria treatment, two additional photographs with focus on bacteria were taken. Clusters were classified as aggregative or snowflake according to the criteria of Pentz et al. (2014) and Ratcliff et al. (2015).

We characterized the mean size of the cells, both within each multicellular cluster and free in the medium (unicellular yeast), using the line in the grid of the Neubauer chamber of length 0.05 mm, and the ImageJ command FitEllipse (Schneider, Rasband, & Eliceiri, 2012). The axis (a & b) of the fitted ellipse was used to estimate the cell area (μm^2^), perimeter (μm), and cell volume (μm^3^) with the formula *V* = 4/3π*a*b2 (Ratcliff et al., 2013). To measure cell area, we used the criterion of covering the whole area of the cell; in nonregular cells, the measured area contained part of outside cells, which was treated as unavoidable error. For each population, we calculated the average of the measurements of three photographs, and for multicellular clusters, at least seven cells per photograph were measured. After identification, each cluster was marked with green spots and counted using the option of particle analysis in the software. The areas of the clusters were calculated based on cell sizes and extrapolated to the whole cluster through the ImageJ software. Cells were counted in all multicellular clusters that we found.

In order to eliminate the bacteria and to check whether multicellular clusters persist after transfers to a new clean media, all populations were grown in YPD medium with a similar antibiotics treatment as indicated before, at 30°C with a shaking frequency of 150 rpm. Then, a small amount was sampled using a sterile inoculated loop, and plated during 48 hr, to obtain colonies. The colonies were grown again in liquid media during 24 hr in the same conditions as before, from which 1 ml was placed in a cryogenic tube with 500 μl of glycerol, and stored at −80°C. Then, multicellular clusters were identified using light microscopy, as indicated before.

### 2.4 Statistics

To compare the clusters across lineages and between treatments, we applied nesting mixed linear models of the form: *y* = μ + *T* + 1|*C* + *e*; where *y* represents the phenotypic data (i.e., area of the cluster, number of cells per cluster, number of unicellular cells per field, and volume of each cell), μ is the phenotypic mean, *T* is the treatment (control and bacteria), *C* is the nested clade (i.e., a random factor of the three subsequent nested divergences), and *e* represents the residual error. Given that the “*C*” factor was not significant at any level, the data were pooled and a linear model (ANOVA) for comparing control with bacteria as single factor was performed. All graphics and statistical analyzes were performed using the lme4 of the R statistical package version 3.0.2 (http://www.R-project.org).

In order to determine whether multicellular clusters appeared randomly across the experimental diversification or if they were associated with the branching pattern of the phylogeny (both, in the control and in the treatment), we performed a phylogenetic signal analysis (sensu Blomberg, Garland, & Ives, 2003), for categorical traits (Rezende & Diniz‐Filho, 2012; Zulqarnain et al., 2016). Briefly, we calculated the minimum number of transitions in character states, at each node of the phylogeny, which accounts for the observed distribution of the character in the tips (Maddison & Maddison, 2000). Then, this magnitude was compared with the median of a randomized distribution (1,000 randomizations were used). A significant phylogenetic signal is then inferred when the observed transition rates fall within the lower tail of 5% of the randomized distribution. Being significant, this outcome implies that the innovation (i.e., the appearance of multicellular clusters) appeared at some point in a given lineage, and affected the derived lineages. If it is not significant, it is concluded that multicellular clusters appeared randomly across the phylogeny (i.e., without reference to the topology; Rezende & Diniz‐Filho, 2012).

## 3 RESULTS

**Figure 2:**
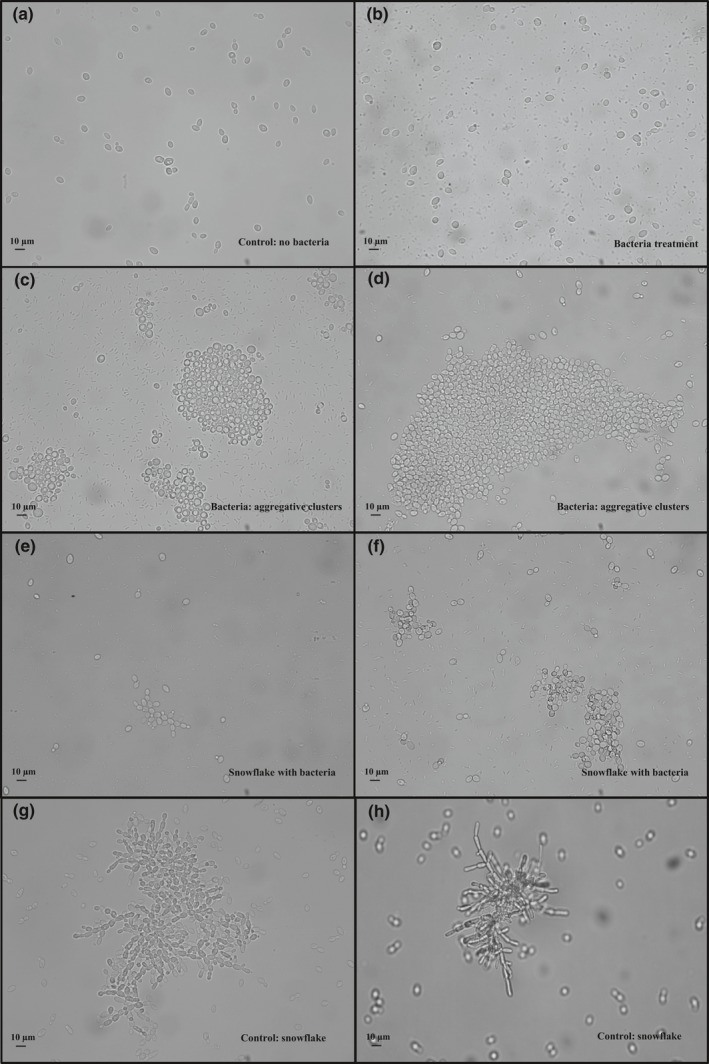
The different forms of uni‐ and multicellular yeasts found in this study. (a) Normal (unicellular) yeasts from the control; (b) normal yeasts in the bacteria treatment; (c and d) aggregative clusters in the bacteria treatment; (e and f) snowflakes in the bacteria treatment; (g and h) snowflakes in the control treatment. No aggregative clusters were found in the bacteria treatment (see Section 3)

In the control phylogeny, no bacteria were identified at any stage of the experiment. Clonal clusters (i.e., “snowflakes,” sensu Ratcliff et al., 2012) were abundant both in the control and in the bacteria treatment, but no aggregative clusters were identified in the control experiment (Table 1; Figure 2). The aggregative clusters identified in the bacteria treatment were large, compact, spherical clusters of tightly attached cells (Figure 2c,d). In contrast, snowflake clusters were smaller and showed the characteristic branching pattern described previously (see Ratcliff et al., 2012; Figure 2e–h).

**Table 1:** Presence/absence of multicellular clusters in the 81 tips of our experimental phylogeny, classified as “snowflakes” and “aggregatives” (see details in Section 2 and Figure 3)

	Control	Bacteria
Snowflakes	78	12
Aggregative	0	20

According to Ratcliff et al. (2013), snowflakes represent clonal multicellularity and aggregatives represent social multicellularity (see Section 1).

**Figure 3:**
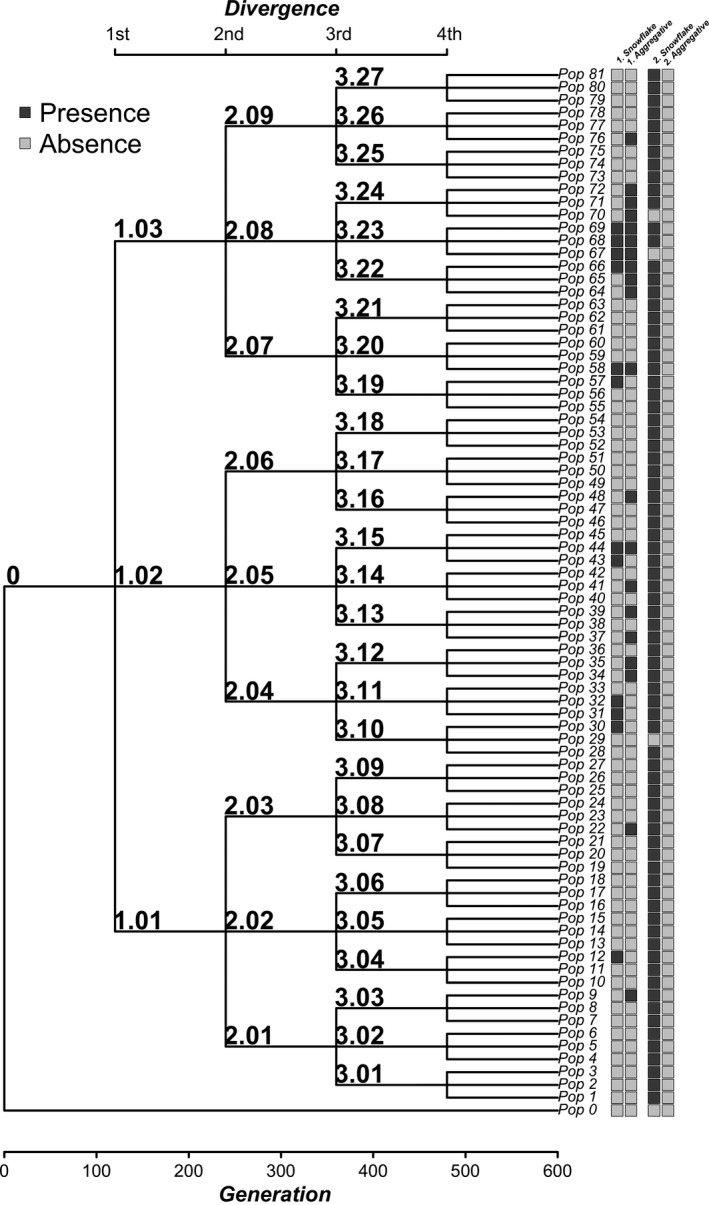
Presence of multicellular clusters (snowflakes or aggregative) in the experimental phylogenies. The columns represents 1—the occurrence of snowflakes and aggregatives under the bacteria treatment and 2—under control conditions. Nomenclature is as in Figure 1. The frequency of each phenotype is indicated in Table 1

**Figure 4:**
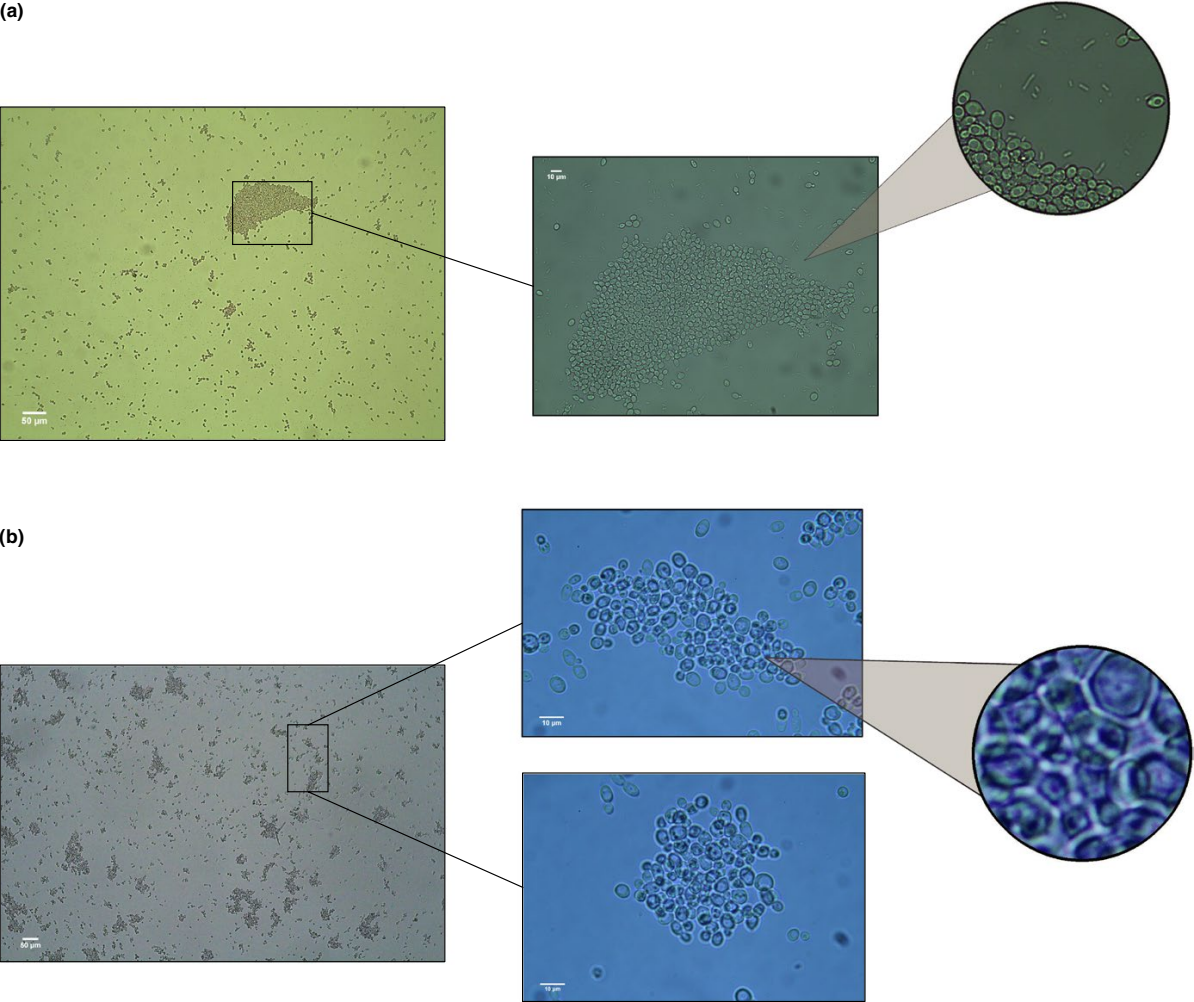
Representative population (Pop 37, according to Figure 1; bacteria treatment) isolated from the bacteria showing the persistence of multicellular clusters after 48 hr of incubation in fresh YPD. (a) The original population with bacteria; (b) the sample isolated from bacteria. In all cases where aggregative clusters were identified, they persisted after transfer to fresh media (see Sections 2 and 3 for details)

At the level of the whole diversification, the control treatment produced 78 tips with recognizable snowflakes (observed number of transitions = 3; median of randomized transitions = 3; *p* = .99), whereas the bacteria treatment produced 12 tips with detectable snowflakes (observed number of transitions = 10; randomized transitions = 12; *p* = .14) (Table 1). Then, none of the transitions were statistically associated with the branching pattern of the diversification. In contrast, aggregative clusters appeared in twenty tips (only in the bacteria treatment), which also showed significant phylogenetic signal (observed number of transitions = 11; median of randomized transitions = 18; *p* < .001; Figure 3). In other words, the distribution pattern of the aggregative phenotype in the resulting lineages is not explained by chance. For instance, all populations originating from lineage 2.08 had aggregative clusters. Similarly, clade 1.02 seems to be associated with a higher occurrence of aggregative clusters, compared with other clades (Figure 3). The aggregative clusters persisted even after eliminating the bacteria from the media using antibiotics and growing them again during 48 hr, suggesting that these are tight associations (Figure 4).

**Figure 5:**
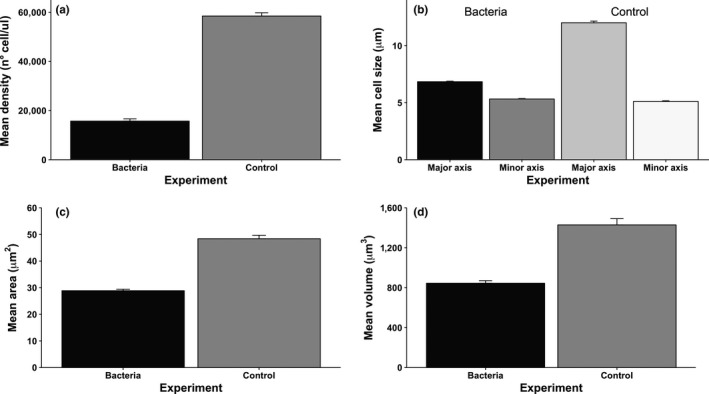
Density and morphometric characteristics of single cells after 600 generations of evolution under bacteria environment, compared with a control. (a) Mean cell density; (b) Mean cell diameter (major and minor axis); (c) Mean cell area; (d) Mean cell volume. Measurements were performed under a Neubauer chamber and optical microscopy (see Section 2 for details). Averages of the 81 populations at the fourth divergence are shown

**Figure 6:**
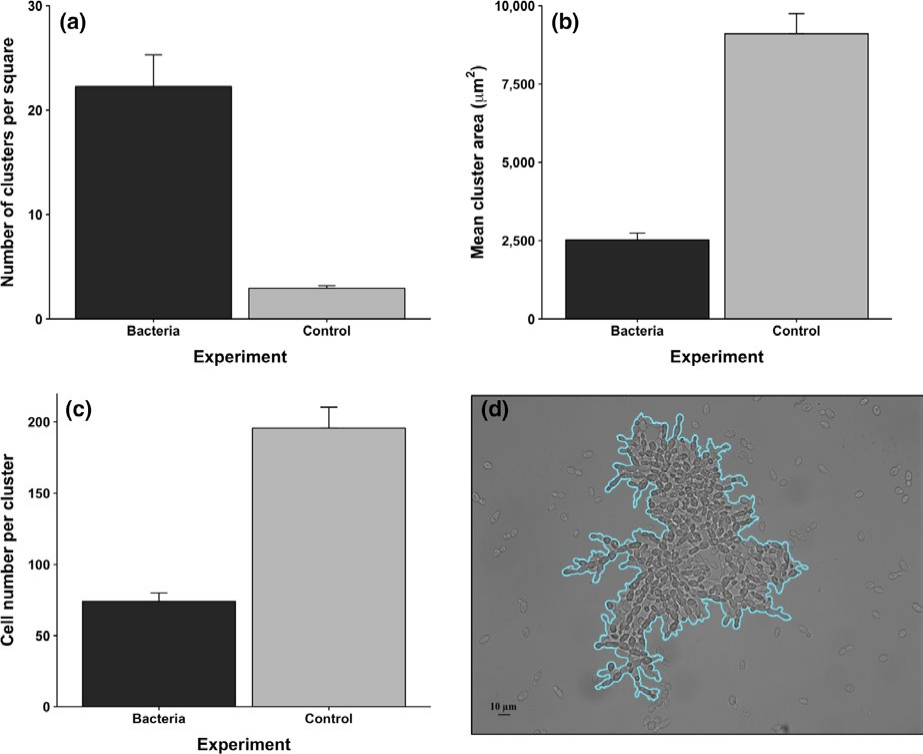
Comparison of snowflakes between the bacteria treatment and the control (there were no aggregative clusters in the bacteria treatment). (a) Density of clusters; (b) Cluster mean area; (c) Mean number of cells per cluster; (d) a detail of the outline used to calculate the area of each clusters. Measurements were taken under a Neubauer chamber and optical microscopy (see Section 2 for details)

In addition to the occurrence of aggregative clusters, we found several differences in cell morphology and density between treatments. Also, a nested ANOVA did not produce significant effects of the hierarchical nesting, which is equivalent to conclude that there were no phylogenetic effects on these variables. First, the density of unicellular yeasts was lower in the bacteria treatment, compared to the control (*F*_1,162_ = 156.92, *p* < .0001; one‐way ANOVA; Figure 5a). Second, under the bacteria environment, cells became smaller compared to the control (Figure 5b–d; comparison for mean cell area; *F*_1,162_ = 109.21, *p* < .0001; one‐way ANOVA; Figure 5c; and volume: *F*_1,162_ = 37.3, *p* < .0001; one‐way ANOVA; Figure 5d). Given that we did not find aggregative clusters in the control, we only compared the characteristics of snowflakes between treatments (Figure 6). Indeed, we found significant differences in the density (Figure 6A; *F*_1,88_ = 14.04, *p* < .0001; one‐way ANOVA), area (Figure 6b; *F*_1,88_ = 29.54, *p* < .0001; one‐way ANOVA) and number of cells per cluster (Figure 6c; *F*_1,88_ = 35.71, *p* < .0001; one‐way ANOVA) of the snowflakes. These differences suggest that snowflakes are more dense (but smaller) in the bacteria treatment, compared with the control.

## 4 DISCUSSION

The bacteria hypothesis for multicellular evolution (“bacteria hypothesis” hereafter) posits that (clonal) multicellularity evolved as a response to bacterial influences, an idea that has been particularly developed for explaining multicellular evolution in animals (Alegado & King, 2014; Alegado et al., 2012; Woznica et al., 2016). This is an appealing concept, as bacteria dominated Earth during at least one billion years before the first eukaryotic organisms (Szathmary & Smith, 1995), and today represent probably the most abundant and ubiquitous living organism (McFall‐Ngai et al., 2013). Hence, it is reasonable to suppose they were involved in the first steps toward multicellular life (Alegado & King, 2014). One of the evidences supporting this idea is elaborated from the capacity of the choanoflagellate *Salpingoeca rosetta* (a close relative to animals) to produce clonal colonies (“collar cells”: feeding structures) in the presence of the bacterium *Algoriphagus machipongonensis* (Woznica et al., 2016). Additional support includes comparative observations of bacteria being involved in organ development in invertebrates or in the settling of larvae in some basal groups of animals (e.g., cnidarians, sponges, bryozoans, and polychaetes, see Alegado & King, 2014; McFall‐Ngai et al., 2013; Woznica et al., 2016, and cited references).

In this study, we followed the alternative strategy of imposing an environment saturated with bacteria (the “bacteria environment,” hereafter) to an experimentally diversifying organism, and to quantify the occurrence of multicellular forms. Our results suggest the bacteria environment promoted the evolution of multicellularity at least in two ways. *First*, it induced the evolution of large and compact clusters that look like defensive associations (sensu Veelders et al., 2010) of genetically diverse individuals (i.e., aggregative clusters) which were not present in the control, and second, it influenced the morphology and abundance of clonal clusters. We also found that aggregative clusters are not transitory; they persist several transfers even when the selective factor was eliminated. Finally, we found that the pattern of experimental evolution of aggregative clusters showed significant phylogenetic signal (i.e., its occurrence is associated with the topology of phylogeny), indicating that only some lineages developed the phenotype.

### 4.1 Social multicellularity as an innovation

Several lines of evidence suggest that aggregative clusters are cases of social multicellularity, where cells come together as a survival strategy (Grosberg & Strathmann, 2007; Ratcliff et al., 2015). This cellular cooperation involves a public good that benefits other cells in the population, which normally has the form of extracellular enzymes, quorum‐sensing molecules or exopolysaccharides (Celiker & Gore, 2013; Kuthan et al., 2003; Veelders et al., 2010). A number of ecological factors could promote cooperation either by simply reducing the amount of nutrients available in the medium (Brockhurst, Buckling, Racey, & Gardner, 2008; Soares & Vroman, 2003), or by directly as environmental insults. Bacteria can provoke direct aggressions to yeast cells by secreting lytic enzymes and cell pathogenicity factors (Bhattacharya, Nagpure, & Gupta, 2007), inhibitory or toxic compounds, such as bacterial fermentation acids (Thomas, Hynes, & Ingledew, 2002), or even by production of antifungal metabolites such as 2,4‐diacetylphloroglucinol (Troppens, Dmitriev, Papkovsky, O'Gara, & Morrissey, 2013). Interestingly, we found that not all lineages in our experimental diversification produced aggregative clusters; in spite of the fact that bacteria equally invaded all. These were persistent clusters that resisted several transfers after the elimination of the bacteria. In yeasts, such associations are mediated by mutations at several genes, which permit cells to strongly stick together; only if both cells express the protein (Smukalla et al., 2008). Hence, social multicellularity could represent an evolutionary innovation in response to bacteria.

### 4.2 What the bacterial environment represents?

We recognize that the approach we followed is unusual and that probably, the standard experimental evolution design (i.e., comparing treatment and control populations) (Garland & Rose, 2009; Kawecki et al., 2012) would have satisfied our primary question. However, we were interested in exploring a novel approach that combined experimental evolution and experimental phylogenies, in order to study diversification in a selective environment (“experimental phylogenetics” sensu Hillis et al., 1992).

What exactly the bacteria environment represents to the yeasts is impossible to define from our data. However, we can speculate what were the main involved factors. We detected at least 23 taxa of bacteria that coevolved with yeasts, and reduced yeast abundance from about 25% at the first divergence to less than 5% at the end of the experiment (Figure S1). Then, (by nutrient depletion) it seems that the bacteria environment imposed competitive conditions to the yeasts, which are the main known effect of bacteria to other unicellular organisms (Bayrock & Ingledew, 2004; Viljoen, 2001). This selective environment promotes efficiency in nutrient acquisition, for which the best strategy in unicellular organisms is to become smaller, increasing the surface‐to‐volume ratio (Alberghina, Rossi, Querin, Wanke, & Vanoni, 2004; Finkel et al., 2010; Vanoni, Rossi, Querin, Zinzalla, & Alberghina, 2005; Yoshiyama & Klausmeier, 2008). Interestingly, we detected such cell size reduction in our bacteria treatment (Figure 5). Another known effect of bacteria on yeasts is acid toxicity, mostly driven by lactic bacteria (Bayrock & Ingledew, 2004; Viljoen, 2001) (e.g., *Gluconobacter freteurii*,* Lactococcus lactis*, and *Weisella confusa*, see Figure S1). Lactic bacteria provoke acid toxicity, which inhibits growth at relatively low concentrations, and is synergistic on the yeasts, when nutrients are scarce (Narendranath, Thomas, & Ingledew, 2001a,b). Finally, there are of course direct toxic effects of bacteria, such as the production of beta lactamase by the pathogenic *Sphingobacterium multivorum* and *Stenotrophomonas maltophilia* which also invaded the experiment and became numerically important at the fourth divergence (Figure S1). Thus, competition and acid toxicity and pathogenicity would have configured the environment where our yeasts evolved.

## 5 CONCLUDING REMARKS: EXPERIMENTAL PHYLOGENETICS REVISITED?

In this study, we applied an experimental phylogenetics approach (“the use of known phylogenies for testing evolutionary hypotheses,” Bull et al., 1993; Hillis et al., 1992; Oakley & Cunningham, 2000; Oakley et al., 2005), to study the origin of multicellularity in a model organism, in the laboratory. Comparative phylogenetics combines experimental evolution and comparative phylogenetic methods, an approach that permitted us to show that multicellular evolution is frequent, it can be clonal or aggregative, and showed that bacteria has a strong influence on its origin. We hope this study will inspire further experimental phylogenetic works where phenotypes are analyzed together with phylogenetic methods, in order to infer how major transitions in evolution could have occurred.

## CONFLICT OF INTEREST

None declared.
