# Supplementary figures and images for: Exploring the evolution of multicellularity in Saccharomyces cerevisiae under bacteria environment: An experimental phylogenetics approach
Source: Ecol Evol. 2018 Apr 15;8(9):4619–30. doi: 10.1002/ece3.3979 (PMC5938455; doi:10.1002/ece3.3979)

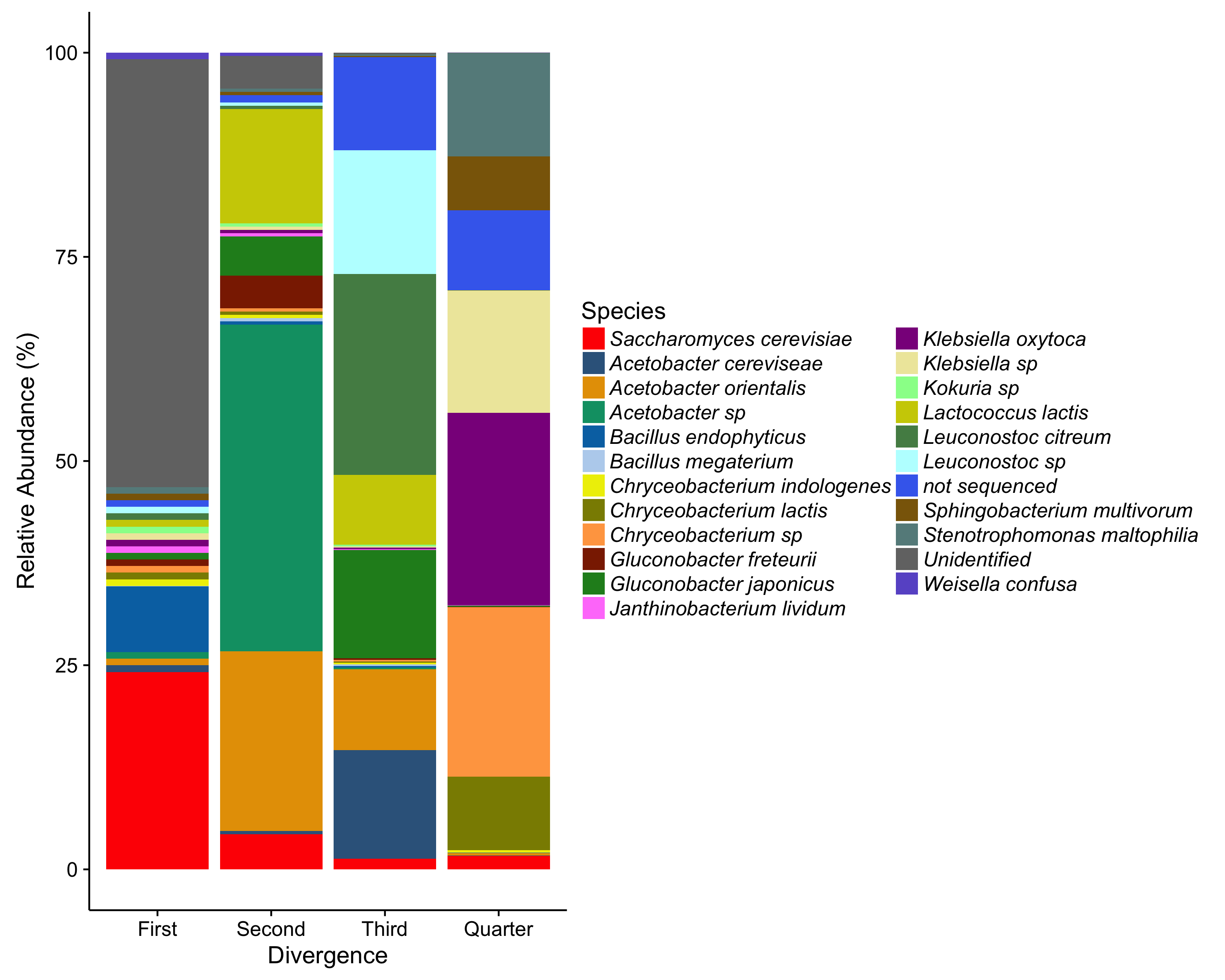

Supplement: Supplementary file 1 [file ECE3-8-4619-s001.png]
