# Supplementary material for: Exploring the evolution of multicellularity in Saccharomyces cerevisiae under bacteria environment: An experimental phylogenetics approach
Source: Ecol Evol. 2018 Apr 15;8(9):4619–30. doi: 10.1002/ece3.3979 (PMC5938455; doi:10.1002/ece3.3979)
Supplement: Supplementary file 2 [file ECE3-8-4619-s002.docx]

Table A1. Abundance of bacteria, of colony-forming units, obtained after plating the media from randomly selected tubes at each divergence (see Methods for details). The nomenclature correspond to the design presented in Fig 1.

| Divergence | Genus / Species | Abundance (cells μL^-1^) | Percentage by Genus/Species |
| --- | --- | --- | --- |
| First | Saccharomyces_cerevisiae | 15000000 | 24.2 |
| First | Acetobacter_cereviseae | 500000 | 0.8 |
| First | Acetobacter_orientalis | 500000 | 0.8 |
| First | Acetobacter_sp | 500000 | 0.8 |
| First | Bacillus_endophyticus_S1 | 5000000 | 8.1 |
| First | Bacillus_megaterium_S4 | 40000 | 0.1 |
| First | Chryceobacterium_indologenes | 500000 | 0.8 |
| First | Chryceobacterium_lactis | 500000 | 0.8 |
| First | Chryceobacterium_sp | 500000 | 0.8 |
| First | Gluconobacter_freteurii | 500000 | 0.8 |
| First | Gluconobacter_japonicus | 500000 | 0.8 |
| First | Janthinobacterium_lividum | 500000 | 0.8 |
| First | Klebsiella_oxytoca | 500000 | 0.8 |
| First | Klebsiella_sp | 500000 | 0.8 |
| First | Kokuria_sp._S2 | 500000 | 0.8 |
| First | Lactococcus_lactis | 500000 | 0.8 |
| First | Leuconostoc_citreum | 500000 | 0.8 |
| First | Leuconostoc_sp | 500000 | 0.8 |
| First | not_sequenced | 500000 | 0.8 |
| First | Sphingobacterium_multivorum | 500000 | 0.8 |
| First | Stenotrophomonas_maltophilia | 500000 | 0.8 |
| First | Unidentified | 32500000 | 52.4 |
| First | Weisella_confusa | 500000 | 0.8 |
| Second | Saccharomyces_cerevisiae | 5400000 | 4.3 |
| Second | Acetobacter_cereviseae | 500000 | 0.4 |
| Second | Acetobacter_orientalis | 27500000 | 22.0 |
| Second | Acetobacter_sp | 50000000 | 40.0 |
| Second | Bacillus_endophyticus_S1 | 500000 | 0.4 |
| Second | Bacillus_megaterium_S4 | 500000 | 0.4 |
| Second | Chryceobacterium_indologenes | 500000 | 0.4 |
| Second | Chryceobacterium_lactis | 500000 | 0.4 |
| Second | Chryceobacterium_sp | 500000 | 0.4 |
| Second | Gluconobacter_freteurii | 5000000 | 4.0 |
| Second | Gluconobacter_japonicus | 6000000 | 4.8 |
| Second | Janthinobacterium_lividum | 500000 | 0.4 |
| Second | Klebsiella_oxytoca | 500000 | 0.4 |
| Second | Klebsiella_sp | 500000 | 0.4 |
| Second | Kokuria_sp._S2 | 500000 | 0.4 |
| Second | Lactococcus_lactis | 17500000 | 14.0 |
| Second | Leuconostoc_citreum | 500000 | 0.4 |
| Second | Leuconostoc_sp | 500000 | 0.4 |
| Second | not_sequenced | 1137000 | 0.9 |
| Second | Sphingobacterium_multivorum | 500000 | 0.4 |
| Second | Stenotrophomonas_maltophilia | 500000 | 0.4 |
| Second | Unidentified | 5000000 | 4.0 |
| Second | Weisella_confusa | 500000 | 0.4 |
| Third | Saccharomyces_cerevisiae | 3483333.3 | 1.3 |
| Third | Acetobacter_cereviseae | 35000000 | 13.3 |
| Third | Acetobacter_orientalis | 26250000 | 9.9 |
| Third | Acetobacter_sp | 500000 | 0.2 |
| Third | Bacillus_endophyticus_S1 | 500000 | 0.2 |
| Third | Bacillus_megaterium_S4 | 500000 | 0.2 |
| Third | Chryceobacterium_indologenes | 500000 | 0.2 |
| Third | Chryceobacterium_lactis | 500000 | 0.2 |
| Third | Chryceobacterium_sp | 500000 | 0.2 |
| Third | Gluconobacter_freteurii | 500000 | 0.2 |
| Third | Gluconobacter_japonicus | 35000000 | 13.3 |
| Third | Janthinobacterium_lividum | 238222.2 | 0.1 |
| Third | Klebsiella_oxytoca | 500000 | 0.2 |
| Third | Klebsiella_sp | 500000 | 0.2 |
| Third | Kokuria_sp._S2 | 500000 | 0.2 |
| Third | Lactococcus_lactis | 22500000 | 8.5 |
| Third | Leuconostoc_citreum | 65000000 | 24.6 |
| Third | Leuconostoc_sp | 40000000 | 15.2 |
| Third | not_sequenced | 30000000 | 11.4 |
| Third | Sphingobacterium_multivorum | 500000 | 0.2 |
| Third | Stenotrophomonas_maltophilia | 500000 | 0.2 |
| Third | Unidentified | 500000 | 0.2 |
| Third | Weisella_confusa | 2000 | 0.0 |
| Fourth | Saccharomyces_cerevisiae | 45475000 | 1.7 |
| Fourth | Acetobacter_cereviseae | 500000 | 0.0 |
| Fourth | Acetobacter_orientalis | 8275000 | 0.3 |
| Fourth | Acetobacter_sp | 500000 | 0.0 |
| Fourth | Bacillus_endophyticus_S1 | 500000 | 0.0 |
| Fourth | Bacillus_megaterium_S4 | 500000 | 0.0 |
| Fourth | Chryceobacterium_indologenes | 7500000 | 0.3 |
| Fourth | Chryceobacterium_lactis | 245000000 | 9.0 |
| Fourth | Chryceobacterium_sp | 562500000 | 20.8 |
| Fourth | Gluconobacter_freteurii | 500000 | 0.0 |
| Fourth | Gluconobacter_japonicus | 5000000 | 0.2 |
| Fourth | Janthinobacterium_lividum | 433333.3 | 0.0 |
| Fourth | Klebsiella_oxytoca | 638966666.7 | 23.6 |
| Fourth | Klebsiella_sp | 405000000 | 14.9 |
| Fourth | Kokuria_sp._S2 | 500000 | 0.0 |
| Fourth | Lactococcus_lactis | 500000 | 0.0 |
| Fourth | Leuconostoc_citreum | 500000 | 0.0 |
| Fourth | Leuconostoc_sp | 500000 | 0.0 |
| Fourth | not_sequenced | 265000000 | 9.8 |
| Fourth | Sphingobacterium_multivorum | 179000000 | 6.6 |
| Fourth | Stenotrophomonas_maltophilia | 343125000 | 12.7 |
| Fourth | Unidentified | 500000 | 0.0 |
| Fourth | Weisella_confusa | 500000 | 0.0 |
